# Adaptation of TECCU App Based on Patients´ Perceptions for the Telemonitoring of Inflammatory Bowel Disease: A Qualitative Study Using Focus Groups

**DOI:** 10.3390/ijerph17061871

**Published:** 2020-03-13

**Authors:** Javier Del Hoyo, Pilar Nos, Raquel Faubel, Guillermo Bastida, Diana Muñoz, Elena Valero-Pérez, Alejandro Garrido-Marín, Pablo Bella, Beatriz Peña, Claudia Savini, Mariam Aguas

**Affiliations:** 1 Gastroenterology Department, La Fe University and Polytechnic Hospital, 46026 Valencia, Spain; delhoyo_jav@gva.es (J.D.H.); nos_pil@gva.es (P.N.); guille.bastida@gmail.com (G.B.); dianalife@gmail.com (D.M.); elenavaleroperez@gmail.com (E.V.-P.); alexgarridomarin@gmail.com (A.G.-M.); 2 CIBEREHD (Networked Biomedical Research Center for Hepatic and Digestive Diseases), Valencia, Spain; 3 Health Research Institute ‘La Fe University Hospital’, 46026 Valencia, Spain; 4 Department of Physiotherapy, University of Valencia, 46010 Valencia, Spain; Raquel.Faubel@uv.es; 5 Joint Research Unit in Biomedical Engineering (eRPSS: IIS La Fe-UPV), 46026 Valencia, Spain; 6 Confederation of associations for patients with Crohn’s disease and Ulcerative Colitis of Spain (ACCU), 28045 Madrid, Spain; secretario@accuesp.com (P.B.); solidaridad@accuesp.com (B.P.); areacientifica@accuesp.com (C.S.)

**Keywords:** inflammatory bowel disease, qualitative research, focus groups, E-health, telemedicine

## Abstract

*Background:* Despite the continuous adaptation of eHealth systems for patients with inflammatory bowel disease (IBD), a significant disconnection persists between users and developers. Since non-adherence remains high, it is necessary to better understand the patients’ perspective on telemonitoring for IBD. Accordingly, this study aimed to adapt the TECCU telemonitoring app to the preferences and needs of IBD patients. *Methods:* A qualitative study was carried out using successive focus groups of IBD patients. Meetings were audio-recorded and a thematic analysis was employed until data saturation was achieved. The first group included patients who had used the TECCU App in a pilot clinical trial, and subsequent meetings included patients with Crohn’s disease and ulcerative colitis recruited from the Spanish Confederation of patient associations. The information collected at each meeting guided consecutive changes to the platform. *Results:* Data saturation was reached after three focus groups involving a total of 18 patients. Three main themes emerged: (1) platform usability, (2) the communication process, and (3) platform content. All participants indicated that TECCU is easy to use, permitting continuous and personalized feedback. According to patients´ perspectives, the platform was adapted to foster a flexible follow-up and shared decision-making using open and safe communication networks. Many participants appreciated the educational elements and, consequently, the app was connected to reliable and continuously updated webpages. *Conclusions:* IBD patients valued the usability and personalized monitoring offered by the TECCU App. Improvements in the messaging system and continuously updated educational content were introduced to address patients´ needs and favor their engagement.

## 1. Introduction

Interest in electronic health (eHealth) as a means to extend access to more complete healthcare services at a lower cost has grown in recent years. In the context of current financial sustainability problems and the excessive load of out-patient consultations faced by health systems, the growing availability of faster and cheaper Internet connections is encouraging the use of eHealth as an instrument to improve the quality and equality of healthcare [1]. Different forms of eHealth interventions have been used, especially to help manage chronic disorders. In the setting of gastrointestinal pathologies, one of the best studied is the use of telemonitoring for inflammatory bowel disease (IBD) [2,3].

IBD is a group of chronic disorders that mainly affect young individuals in the prime of their personal and professional development. It is related to high levels of school and work absenteeism, disability [4], interference with social activities, and impaired health-related quality of life (HRQoL) [5]. Therefore, IBD has a significant medical, social, and financial impact. Moreover, the burden and healthcare costs associated with its management continue to rise [6,7], which currently makes it one of the most expensive gastrointestinal conditions [8]. In an attempt to overcome these problems, web-based telemonitoring systems have been safe and feasible tools that empower IBD patients with the capacity to better control their disease, producing fewer outpatient visits, hospital admissions and, thus, potential cost-savings [9,10,11].

However, most eHealth applications do not pass beyond the pilot stage [12], mainly due to barriers that hinder their implementation in daily practice and policy. There is often insufficient technical training for healthcare providers and patients, and there are concerns about the privacy of health data as well as confusion regarding the reimbursement system for telehealth services [13]. Moreover, and despite the potential cost-savings of eHealth in the IBD setting [14], the efficacy of such approaches in terms of disease outcomes is not consistent across studies [9,10,15,16,17,18,19]. Remarkably, the attrition rates reported are still high despite the continued adaptation of web platforms and the evolution of mHealth over the last decade [9,17,18,19,20].

In this sense, it is important to note the disconnection that exists between the users and the developers of eHealth systems. The majority of the current apps developed to manage IBD have lacked the input of medical professionals during their design [21]. In other clinical settings, the patients´ point of view has been used to evaluate the acceptance of new eHealth systems. This consideration is essential because understanding the factors that influence user acceptance allows us to address their requirements and to promote the use of these programs [22,23,24,25,26]. 

Information and communication technologies have evolved to provide easier and faster data transmission through new communication services, which are especially useful in remote areas [27]. However, adherence to distance care is not only based on technological advances. The development of eHealth allows an efficient delivery of health services to face the growing healthcare costs and the need to empower patients to manage their selfcare. These new tools support the integration of multidisciplinary care for patients with chronic diseases [28]. However, the design of new telemedicine programs should also consider that patients with different chronic diseases prefer to employ useful and high-quality systems, which allow them to cooperate with healthcare providers [22,23,24,26]. Accordingly, incorporating patients in the design, development, and implementation of eHealth will potentially improve patient engagement with a remote follow-up [29]. Recent efforts have been made to assess patient perceptions and other issues associated to telemonitoring adherence in IBD [30,31].

Our research group developed a web-based telemonitoring program known as TECCU (*“Telemonitorización de la Enfermedad de Crohn y Colitis Ulcerosa”* or Telemonitoring of Crohn’s Disease and Ulcerative Colitis) [32]. A previous pilot trial suggested that TECCU is a safe strategy to improve health outcomes for IBD patients [19] with a high probability of being more cost-effective in the short-term than standard and telephone care [14]. Although non-adherence was relatively low in our trial, we hypothesized that the incorporation of new technologies over time and the usability of the platform over longer follow-up periods could modify the patient engagement we observed in our pilot study. As such, we performed a qualitative study using a thematic analysis approach to explore IBD patients´ preferences and needs for distance care. This study evaluates the patients´ perceptions in order to adapt the TECCU telemonitoring app, which provides a framework for future modifications of eHealth tools for IBD management.

### Structure of the Paper

In this article, we detail the methodology used to run the successive focus groups and analyze the information collected. The recordings were classified into codes, categories, and major themes, which guided further adaptation of the TECCU app. In the results section, the characteristics of patients who participated in the study are described, and the emergent themes, categories, and codes, as well as their relationships, are presented in a coding tree. According to each emergent theme, we detail the patients´ needs that were recorded and we illustrate the related platform changes. Lastly, in the discussion, we compare our findings with those reported in the literature, providing a conclusion and recommendations for future research.

## 2. Materials and Methods 

### 2.1. Study Design

We performed a qualitative study using focus group discussions to explore the experiences and perceptions of patients with IBD who used the TECCU telemonitoring web platform. This study is embedded within a clinical trial designed to evaluate the impact on health outcomes of the TECCU web platform for telemonitoring patients with complex IBD in comparison with telephone and standard care [19]. Data was collected to gain a more complete understanding of the TECCU app’s usability and the patients´ needs regarding the app. Focus groups were established in accordance with the Krueger and Casey guidelines [33], and the interviews were audio-recorded. Three different researchers (JH, MA, RF) analyzed the recordings independently and inductively, adopting a thematic analysis approach to obtain different codes, categories, and themes until thematic saturation was achieved. We followed the consolidated criteria for reporting qualitative research (COREQ) 32-item checklist [34], and this information was used to guide consecutive changes in the web platform.

### 2.2. Working Process and Patient Selection

The successive focus groups allowed us to collect, transcribe, code, and analyze the perceptions and needs associated with the use of the TECCU app. The information was gathered from a purposive sample of patients from the IBD Unit at La Fe University Hospital who used the platform in the pilot trial, and from other patients not included in the trial that were contacted through the confederation of associations for patients with Crohn’s disease and ulcerative colitis of Spain (ACCU). Patients who participated in the pilot trial were contacted via e-mail and telephone, while those from ACCU were contacted via e-mail using the organization’s internal communication channels. We aimed to include four to eight patients in each group, which is in agreement with general recommendations [35,36].

**Figure 1:**
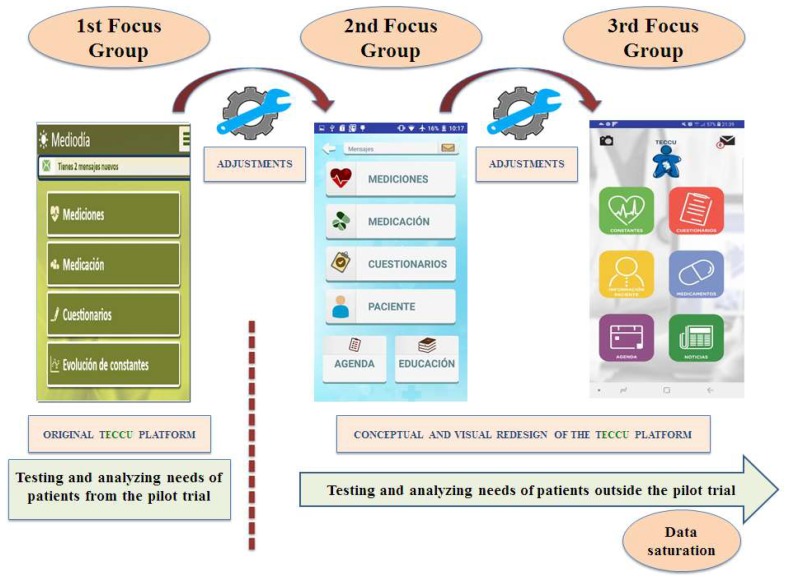
The workflow followed to guide the continuous adaptation of the TECCU platform.

Each focus group lasted for 90 to 120 minutes, and they took place at facilities adapted for this purpose outside the hospital. The different codes, categories, and themes obtained by each researcher with this method were then triangulated before holding the following focus group. All these data were used to guide new adjustments to the web platform, which were then evaluated by other IBD patients in the subsequent focus groups. This process of focus group interviews, researcher analysis and triangulation, and platform adjustment was repeated until no new themes emerged (see Figure 1).

### 2.3. First Focus Group with Patients from the Pilot TECCU Trial

The first focus group was held at the University of Valencia on 14 June, 2018. The participants were adult IBD patients (≥ 18 years of age) who used the NOMHADCHRONIC web platform to monitor disease activity after initiating treatment with an immunosuppressant or biological agent in a pilot study developed by our group [19]. A purposive sampling method was used to enroll patients previously followed with the platform, independent of their adherence to telemonitoring. The facilitator who conducted the focus group (RF) was an epidemiologist skilled in carrying out focus group interviews in collaboration with an assistant (MP) who took notes on the most important themes that emerged from the meeting. Both the facilitator and the assistant had no previous contact with the patients.

The qualitative data registered from this focus group was coded and analyzed in order to introduce the relevant changes into the web platform. The new platform, modified on the basis of these patients´ perceptions, was evaluated in two further focus groups involving IBD patients who were not participating in the pilot trial.

### 2.4. Second and Third Focus Groups with IBD Patients Outside the Pilot Trial

These second and third focus groups were carried out in collaboration with the ACCU, apatients´ organization with more than 8,000 members distributed over 36 regional groups across the country. The interviews were moderated by a psychologist who belonged to the organization (VA) with the assistance of two facilitators (BP, CS). The patients that participated did not know either the TECCU researchers or the psychologists moderating the interview.

The new web platform, adapted on the basis of the information obtained in the first focus group, was presented to the second focus group of IBD patients on 23 October, 2018. We evaluated the experiences and perceptions of this second group of patients regarding the newly adapted platform. The issues they raised were used to guide further changes to the platform, which were then subsequently tested by another group of patients, purposively sampled in a third focus group that was held on 27 February, 2019.

### 2.5. Data Collection

The Krueger and Casey recommendations [33] were followed to guide the questions that were discussed by the focus groups. We explored the follow-up needs of patients related to the content, structure and use of telemonitoring, and the TECCU web platform [37].

We designed a group of open-ended questions regarding: 1) the patients´ expectations about the use of a telemonitoring platform; 2) the ability of the TECCU platform to address the most important problems related to disease management; and 3) the interaction of patients with the TECCU platform. The questions were designed based on the recommendations of two gastroenterologists (JH, MA) and two facilitators (BP, CS).

The guidelines for the questions used in the focus group are presented in Appendix A.

### 2.6. Data Coding and Analysis

All the group discussions were recorded and then transcribed by JH. Three researchers (JH, MA, RF) analyzed this information individually after each focus group using a thematic analysis approach [38]. Subsequently, the emergent themes and the data saturation was assessed collaboratively by all three researchers. 

This process started by reading the transcripts to become familiar with the data. Subsequently, the researchers used ATLAS.ti version 8.4.14 to encode the information obtained in successive cycles, according to the general coding recommendations [39]. In the first cycle, each researcher developed the initial codes and categories individually, which were then refined in the ensuing cycles. By analyzing the different codes and the major categories obtained, emerging themes were defined and then triangulated between the researchers to reach an agreement on the interpretation of the information. This is a process that was performed until data saturation was achieved. Moreover, we included relevant quotes that illustrated the findings obtained. The different codes, categories, and themes were grouped into a coding tree chart that illustrated the patients’ requests and preferences for telemonitoring. Lastly, patients were asked to provide their feedback on the emergent themes obtained for correction if needed.

### 2.7. Ethical Considerations

The study was approved by the ethics committee at La Fe University Hospital, Valencia, Spain. All the participants were provided with project information and gave their informed consent to participate. They were all given a numerical ID to protect their anonymity during the identification of transcripts and the reporting of the data.

## 3. Results

We ran successive focus groups until thematic saturation was achieved and this point of saturation was reached after holding three focus groups. To recruit the participants to this study, a total of 12 patients who used the TECCU app in a pilot study and 32 patients associated with the ACCU were contacted. From the 12 patients participating in the TECCU study, four patients were included in the first focus group. The other eight patients were not included because six of them could not attend the meeting and another two did not respond, such that no patients actually refused to participate. In terms of the 32 patients contacted through the ACCU, 14 patients participated in the focus groups (seven in each). Of the remaining 18, seven failed to participate because they could not attend the meeting, 10 did not respond, and one refused to participate. Thus, a total of 18 patients (four from the pilot study and 14 who were not involved in the trial) participated in the three focus groups.

### 3.1. Demographics

The demographics and characteristics of the whole group of participants are shown in Table 1. The median age of the patients was 37.5 years (range 20–63), and the patients included were diagnosed with CD (n = 10) or UC (n = 8), with a median disease duration of 18 years (range 3–39). The majority of the participants (12 out of 18) were considered to be in remission. In terms of the educational level, 13 of the 18 patients had completed university studies, and 15 of them accessed the Internet daily on their mobile phone.

### 3.2. Emerging Themes

**Figure 2:**
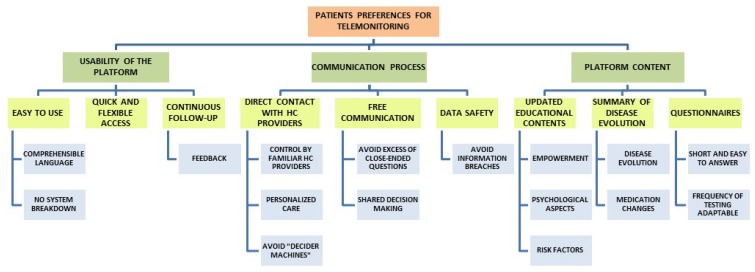
Coding tree representing the codes, categories, and themes that emerged in the focus groups.

The analysis of the information recorded in the consecutive focus groups highlighted the IBD patients’ perceptions of telemonitoring. This provided insight into the patients’ preferences of the ideal features for a telemonitoring platform and their experiences in using the TECCU app. The main themes that emerged were: (1) the usability of the telemonitoring platform, (2) the nature of the communications through the web platform, and (3) the content the platform should include. The emergent codes, categories, and themes were represented in a coding tree chart (Figure 2), and we then modified the platform accordingly. The proportion of patients who agreed with the themes and categories that emerged in the meetings is indicated in Table 2.

### 3.3. Usability of the Telemonitoring Platform

#### 3.3.1. Patients’ Perceptions and Needs Regarding Usability

All patients reported that a telemonitoring platform to control IBD would be useful if it were easy to use. They recommended not including too many questionnaires and they wanted the information in the app to be expressed in a clear and understandable manner. Some patients also referred to the importance of maintaining the system continuously operative by avoiding any collapse of the system and resolving such problems efficiently if they occur.

“When I access the platform I am prepared to answer four or five essential questions but not a long questionnaire that I barely understand… I want to know that if I send a message someone will read it and that the system won’t collapse.”[P05]

Moreover, all the patients wanted a flexible system that adapted the frequency of accessing the web platform to their particular disease evolution. During a flare-up, they would like to contact their healthcare providers as quickly as possible and, when in remission, they would like to reduce the complexity and frequency of contact. Most of the patients agreed that they would use the platform approximately once a week when the disease is active and once a month when in remission.

“When I feel fine I won´t spend more than 5 minutes using the app… I don´t want to be thinking about my illness all the time… and when I feel worse, I´d like to be able to contact my nurse or doctor quickly.”[P09]

In this sense, even if the patients wanted to adapt the follow-up schedule to their disease course, most of them (13 out of 18) wanted to feel in control and that the platform is safe.

“For me it´s important to feel that there is someone else on the other side… You know… I think it´s very important to be sure that my doctors will look at my messages, or the results of my blood tests or the calprotectin results…”[P04]

#### 3.3.2. Improvements in the Usability of the TECCU App

In the first focus group with patients who had used the platform in the pilot study, all of them thought that the TECCU was simple to use and that it didn’t take up too much of their time. They liked the “comments” section, where they could freely indicate their health status and concerns. In terms of connectivity, one patient referred to connection problems at the beginning but that the technical team resolved these. Despite the good usability profile, three out of four patients agreed that the platform included a group of questions that were reiterative and sometimes boring. Thus, the frequency of the test questionnaires was adapted to each case and the messaging system was always open according to the patients´ needs.

**Figure 3:**
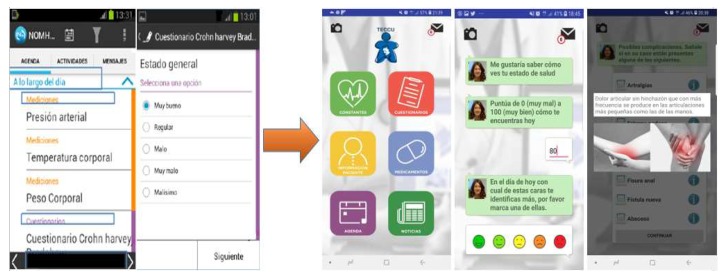
Adaptations to the TECCU platform design to improve usability.

In the second and third focus groups, the patients made similar comments about the usability of the platform, indicating that it was fast, intuitive, and that they also valued the messaging system. However, many of them still noted that some of the content was not clear or easy to read. Moreover, they missed some feedback from the questionnaires and they did not understand the meaning of some of the specific terms. To address these comments, we reassessed the readability of the platform’s components and adapted the patient-reported outcomes (PROs) [40,41,42,43,44]. Beyond the adaptation of the self-testing frequency, we also incorporated the option to self-evaluate disease activity, quality of life, and issues related to medication to a conversation format (Figure 3). In this sense, our group is currently working on incorporating artificial intelligence functionality to create a chat-bot designed for IBD patients.

### 3.4. Characteristics of the Communication Process through the Web Platform

#### 3.4.1. Patient Perceptions and their Communication Needs

In the three focus groups, all the patients agreed that the ideal platform should facilitate efficient communication with healthcare providers. In fact, most of them noted that it would be preferable to establish communication with their usual providers in order to receive personalized care, and some of them suggested forms of distance care other than telemonitoring. For these reasons, many patients found it strange to make decisions regarding adjustments to their medication only based on a computer algorithm.

“The most important thing is being able to communicate with professionals. It´s better when I talk to my doctor or nurse because they understand me and they know what to do when I have a flare-up… Sometimes it might be better if a videoconference were available.” [P03]

“I don´t think that an algorithm fits my specific case…” [P05]

The majority of patients (14 out of 18) preferred to have the opportunity to communicate through an open communication protocol, allowing them to describe their symptoms, feelings, and concerns about their disease in depth, and to discuss the medication they need. Most of them accepted answering some closed questions. Yet, the majority gave more importance to the messaging system and to share their decisions with their healthcare providers.

“I feel safer talking to them about the best treatment option for me when I´m not well, mainly when I know that they will respond if they see something´s wrong.” [P13]

“One possibility would be to not always repeat the same questionnaires. I prefer to choose the questions that are best adapted to me at each moment.” [P07]

In any case, four patients did not want to make decisions themselves in order to avoid becoming ill. They considered it better that the doctors decide the best options for them to manage their disease.

“I think that my doctor knows the best treatment for me…I don´t want to make bad decisions regarding my medication, so if I can contact my doctor faster through the app to know what I should do then that´s positive.”[P02]

In addition, many patients indicated their concerns about the potential security breaches related to contact through the Internet or non-secure servers. All the participants wanted to be sure that their health data will be treated in confidentiality by their usual caregivers.

“I won´t talk about my illness if I don´t trust the platform… I mean… You know when you find a secure link in the Internet? This is important… Maybe it´s better not to use the Internet.”[P17]

#### 3.4.2. Improvements in the Communication Process through the TECCU App

Patients from the first focus group indicated that they preferred to communicate with their healthcare providers through the platform’s messaging system instead of answering questionnaires. For them, it was very important that they received a quick response through the TECCU app and they felt safer in the knowledge that doctors would be aware of any eventual complications. Especially during periods of remission, they preferred to use some form of remote control rather than outpatient visits.

**Figure 4:**
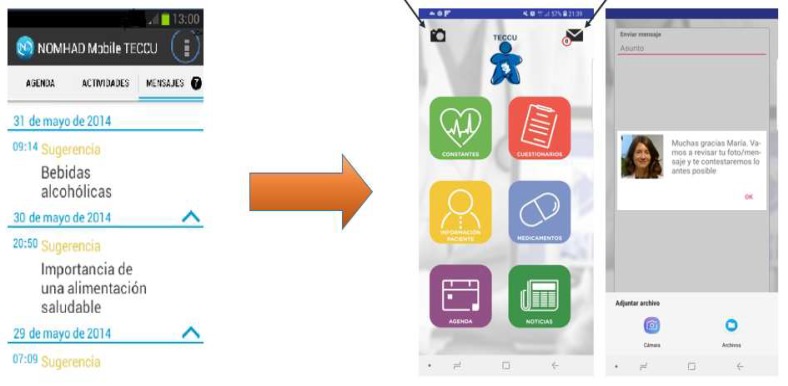
Adaptations to the communication process in the TECCU app.

In accordance with the issues raised by the first focus group, patients from the other two groups preferred to use the messaging system, even though they also raised the possibility of sending images if there were guarantees about the security of the communication process. They also wanted the app to give continuous feedback from the healthcare providers by avoiding decision-making processes driven by algorithms incorporated into the platform itself. In this regard, we included the option to send images using the phone’s camera and the system will return automatic messages adapted to the patients’ needs (Figure 4). Nevertheless, the platform was designed so that the nurse or doctor will provide a personal response to the patients’ problem in 24 to 48 hours. Moreover, the platform uses the Secure Sockets Layer (SSL) to ensure confidentiality during data transmission, and the information received will be stored on a secure server located in the European Union.

The links to the messaging system and the camera to send images are marked with arrows.

### 3.5. Content that the Platform Should Include

#### 3.5.1. Patient Perceptions and Their Needs in Terms of Platform Content

Many patients wanted to have access to up-to-date content regarding their disease and medication. These patients considered that better access to reliable information could better empower them to self-manage flare-ups or complications in collaboration with their caregivers. Furthermore, they wanted to receive advice as to how they should deal with the events that might occur as their disease evolves, and even recommendations about diet and risk factors that could lead to a flare-up.

“I search the Internet when I want information, so it would be good to have access to reliable news about new medications or the illness through the platform …I’m not saying that I can control my illness by myself but maybe, if I’m better informed about what might make me feel worse I could avoid these things.” [P01]

“For me it´s very important to know what I should eat in order to feel better and to help stave off my illness. This is never clear to me and, thus, it would be interesting if the platform included information that dispelled false beliefs about food.” [P15] 

“… and what if I have acute gastroenteritis? I would feel calmer if I could get some advice to confirm that it is not a flare-up [of my disease] or something similar…” [P10]

However, there was a subgroup of four patients that preferred not to consult the Internet but, rather, they wanted to be informed through their healthcare providers.

“I prefer not to look for information by myself. I don´t know exactly what´s right or wrong and I try to address any doubts I have directly through my doctor or nurse.” [P06]

Beyond self-management, all patients would like to have access to other specialists through the platform and they mentioned the possibility of addressing the psychological impact of IBD.

“Would it be possible to contact a psychologist? I don´t know…This illness has a strong effect on you and I´d like to have access to other professionals if necessary.” [P02]

Most patients accepted the incorporation of short questionnaires to self-test their health, while still prioritizing the use of open communication tools. Moreover, many patients considered it interesting to have access to calprotectin home-tests to improve self-control. Like accessing the platform according to their particular disease evolution, all patients wanted to adapt the frequency of self-testing to their particular disease activity at any given time.

“I don’t want too many questions in the app. I mean… I understand that doctors need to use some questionnaires to classify the severity of the disease but, if I need help, I want the app to be easily accessible so that I can contact my doctor.” [P12]

“I would like to count on calprotectin kits at home to improve disease control.” [P10]

In addition, and in order to be more proactive, 12 out of 18 participants were interested in platforms that show a summary of their disease evolution and changes in medication over time, which could potentially improve their control of the disease.

“I´d like to have access to a summary recording of the flare-ups that I have had and to the changes in my medication. Maybe I could use it to see what worked before and to inform other doctors if I have to go to the emergency department.” [P18]

#### 3.5.2. Updates in the Platform Content

During the first focus group, all the patients agreed that the educational content of the platform was interesting but that they also missed having faster updates of that content. Accordingly, the app has been connected to reliable webpages of nationally accredited IBD units and patient associations. These pages are continuously updated with news related to different aspects of the disease and medications, including items about the psychological management of the disease, diet, and the risk factors associated with disease activity in accordance with the patients’ desires.

**Figure 5:**
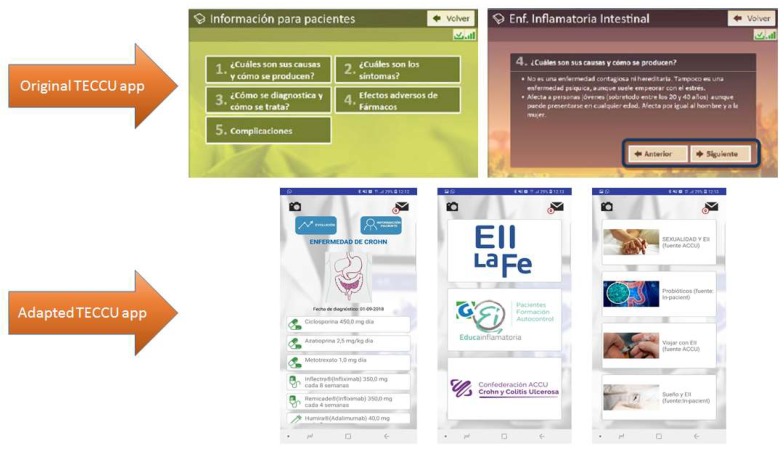
Updates to the educational contents and the summary of the patients’ disease course in the TECCU app.

Patients from the second and third focus groups appreciated these adaptations to this educational content. Yet, they also indicated that it was important to make the information included in the questionnaires more comprehensible and practical. In this sense, we adapted simple and validated patient-reported outcomes (PROs) [40,41,42,43,44], and included some information to illustrate in text and images the meaning of specific terms like aphtha, arthralgia, etc. Moreover, the app was redesigned to include a section that contained a summary of the patients’ disease evolution and the medications prescribed (Figure 5).

## 4. Discussion

In this study, we used focus groups to direct the modification of the TECCU app to the perceptions and needs of patients with IBD. The importance of this work lies in the potential benefit of incorporating patient opinions into the design and validation of telemonitoring platforms to improve their engagement with remote follow-up systems. Currently, telemedicine is suffering from ‘pilotitis’ with many eHealth projects failing to scale-up, and enter daily practice and policy. Among the barriers to the wider implementation of eHealth, there is a disconnection between the users and developers of eHealth systems with attrition rates still high despite the continued adaptation of web platforms over recent years. The patient perspectives reported in this paper could orientate the design of future apps for IBD management, particularly since a few studies have examined IBD patient preferences about telemonitoring [30,31,45].

During the successive focus groups, three major themes emerged: the usability of the web platform, the communication process, and the platform content. Importantly, some of the themes, categories, and codes that emerged were similar to those identified in other qualitative studies on IBD patients [30,31,45]. Different models have been implemented to identify the factors related to the patients´ intention to use new eHealth tools. In other medical settings, patients prefer to employ useful and easy-to-use systems, which allow them to cooperate better with healthcare providers through flexible follow-ups [22,23,24,26]. These factors are largely represented in our findings, which supports the reproducibility of the results presented in this article.

In relation to the usability of the platform, patients wanted to have quick and flexible access for telemonitoring apps. They were reluctant to answer excessive close-ended questionnaires, and they preferred to receive continuous follow-up and personalized feedback adapted to their particular evolution, which is similar to patients in other studies [30,45]. In this sense, we maintained the questionnaires to classify the patients’ health status, but we also adapted simple and validated PROs previously used for telemonitoring of IBD patients [17,18,40,41,42,43,44]. Additionally, some specific terms were presented through text and images so that they were more easily understood.

The frequency of self-testing was adapted to the particular evolution of each patient. Moreover, the TECCU app was designed to combine the results of objective biomarkers with the PRO values, which is in agreement with recent recommendations to improve accuracy in predicting disease activity [31,46]. Furthermore, the questionnaires were redesigned in a conversation format. Our group is currently working on the incorporation of artificial intelligence to develop a Chat-bot oriented toward IBD patients. This is an innovative approach and there are currently some promising projects underway in the IBD field [47,48]. Due to the relapsing course of this disease, such a tool could potentially address the frequent monitoring needs of these patients.

Regarding the interaction with healthcare providers, the study participants also remarked that the telemonitoring app must promote free communication. They wanted to connect with their usual caregivers and rejected the idea of changing medication based only on “machine-made decisions.” These findings are striking when considering that most telemonitoring apps designed for IBD patients are based on systems of alerts and preformulated action plans derived from the patents’ responses to self-reported questionnaires [9,10,17,18,19]. In this sense, the perceptions of the patients remind us of the importance not to consider telemonitoring as an isolated tool to replace in-person visits, but, rather, an adjunct to classic follow-up procedures that potentially optimize healthcare resources [18,49].

Although the initial TECCU app was conceived as a support tool to be combined with standard care [19], the messaging system was redesigned and incorporated imaging elements to improve bidirectional and flexible communication. Even if TECCU is an asynchronous “store and forward” technology, it is designed to give a quick response (24 to 48 hours), which allows shared decision-making continuously supported by healthcare providers. According to the concerns about the privacy of telemonitoring [50], our platform incorporates protocols to ensure confidentiality during health data transmission, which will be stored on a secure server.

Concerning the design of the platform content, we took into consideration that most patients access the Web to obtain information about their disease and medication. They want to visit reliable pages, yet the quality of more than half of the IBD-related websites is fair to poor and most of them are difficult to understand for a substantial proportion of patients [51,52,53]. In this regard, the education section of the TECCU was modified to include direct access to corporate websites that belong to the IBD units participating in the TECCU project, as well as the ACCU Spanish website [54]. These pages contain reliable information and the TECCU incorporates a subsection of news maintained by healthcare providers according to the patients’ interests in the form of practical advice or “false beliefs about IBD.”

Alternatively, many patients would like to participate in self-control and, in fact, most of them wanted to use calprotectin home-tests to better identify disease activity at any time. As noted before, the TECCU app is designed to combine PRO values with those of the calprotectin home-tests, which is in good agreement with the calprotectin measures obtained through ELISA laboratory tests [55].

The patients´ needs and perceptions identified in this study were obtained from successive focus groups, not only from participants of the TECCU pilot study, but also from patients selected by the ACCU, which is an independent organization that represents a wide proportion of patients across the country. To obtain a wide variety of views and achieve data saturation, the recommended number of focus groups needed depends on factors related to the complexity of the topic studied, the homogeneity of the patient sample, the purpose of the study, and the analyst’s categorization style [56]. In this case, three researchers (JH, MA, RF) analyzed the transcripts obtained from each focus group to triangulate the data before holding the next one in order to introduce the patient-guided changes that were evaluated in the subsequent meetings. This process was continued until no new themes emerged, which is a point of saturation that was achieved after three focus groups in accordance with the majority of guidelines [33,35,36].

There are some limitations to this study that must be considered. In the first focus group, we only recruited four patients from the pilot study and, while this is sufficient to meet the general recommendations of 4 to 12 participants per group [35,36], more patients may have provided more diverse opinions. Nevertheless, the information obtained from initial focus groups widely agreed with those obtained in the other two groups with patients recruited by the ACCU. Moreover, the sample size in these other two focus groups was adequate to reach thematic saturation. Lastly, the use of a purposive sampling method could hinder the generalizability of the data obtained. Yet, as we included different populations of patients, many emerging themes agreed with those generated in other studies [30,31,45]. This approach also allowed us to maximize the information gained from key informant patients, which guided further adaptations in the TECCU app.

## 5. Conclusions

In conclusion, different patient profiles valued the TECCU app as a quick and easy-to-use platform. Previous studies already reported that the usefulness and ease of use of the platform are factors related to patient engagement with eHealth technologies. However, this paper also highlights the importance of adapting telemonitoring systems for continuous and personalized feedback. The patients´ perceptions indicate that platform design should favor shared decision-making with healthcare providers through an open and safe communication process. In this sense, the original messaging system was redesigned and incorporated images to improve bidirectional, flexible communication. Moreover, the frequency of self-testing was adapted to the participants’ requests, and the app was connected to high quality and continuously updated webpages. 

This paper provides a research framework for the future adjustment of eHealth tools in IBD. To favor user adherence to telemonitoring, future programs should adapt their follow-up schedules to each moment in their disease evolution. The design of simple and easy-to-use platforms for such a heterogeneous group of disorders is particularly challenging. Furthermore, the integration of high quality and updated educational content is an option that could potentially empower patients willing to use them. Beyond these recommendations, the development of future eHealth programs should continuously evaluate patients´ acceptance in order to ensure a patient-centered design. Nevertheless, it is important not to consider telemonitoring as an isolated tool to replace in-person visits but, rather, represent a new model that potentially optimizes healthcare resources in combination with classic follow-up procedures.

## Figures and Tables

**Table 1:** Demographics and characteristics of patients.

	Sex	Age (Years)	Disease Profile	Disease Duration (Years)	Active/ Remission	Education	Internet Use
**Patients from the pilot TECCU study**							
**P 01**	Female	20	UC (E3)	3	Remission	University	Daily
**P 02**	Male	60	CD (L3)	39	Active	Secondary	Weekly
**P 03**	Female	38	UC (E3)	16	Remission	University	Daily
**P 04**	Female	63	CD (L1)	24	Remission	Secondary	Weekly
**Patients outside the pilot TECCU study**							
**P 05**	Female	42	UC (E3)	28	Remission	University	Daily
**P 06**	Male	43	UC (E3)	4	Active	University	Daily
**P 07**	Male	34	CD (L1)	3	Active	Secondary	Daily
**P 08**	Female	33	UC (E2)	29	Remission	University	Daily
**P 09**	Male	23	CD (L1)	4	Remission	University	Daily
**P 10**	Male	37	UC (E2)	18	Active	University	Daily
**P11**	Female	62	CD (L3)	30	Remission	Secondary	Weekly
**P12**	Female	41	CD (L2)	7	Remission	University	Daily
**P13**	Male	22	UC (E3)	5	Remission	University	Daily
**P14**	Male	36	CD (L1)	18	Active	University	Daily
**P15**	Male	52	CD (L1)	25	Remission	University	Daily
**P16**	Female	37	UC (E2)	23	Remission	Secondary	Daily
**P17**	Male	42	CD (L3)	27	Remission	University	Daily
**P18**	Female	29	CD (L1)	10	Active	University	Daily

Abbreviations: UC, Ulcerative colitis. CD, Crohn´s disease.

**Table 2:** Agreement of patients with the themes and categories that emerged.

Theme	Category	Number of Patients who Agree n (%)	Study ID of Patients who Agree
**Platform Usability**	Easy to use	18 (100)	01 to 18
	Quick and flexible access	18 (100)	01 to 18
	Continuous follow-up	13 (72.2)	01, 02, 04, 07, 08, 09, 10, 12, 14, 15, 16, 17, 18
**Communication process**	Direct contact with HC providers	18 (100)	01 to 18
	Fluid communication	14 (77.8)	01, 03, 04, 06, 07, 08, 09, 10, 11, 12, 13, 15, 16, 18
	Data safety	18 (100)	01 to 18
**Platform Content**	Updated educational content	14 (77.8)	01, 02, 03, 05, 07, 08, 09, 10, 12, 13, 15, 16, 17, 18
	Summary of disease evolution	12 (66.7)	01, 03, 05, 06, 07, 09, 10, 12, 13, 16, 17, 18
	Questionnaires	11 (61.1)	01, 02, 03, 06, 08, 10, 11, 13, 15, 16, 17

## Appendix A

Guide of questions performed in the successive focus groups:

Good evening and welcome. Thank you for your time to talk about your preferences regarding IBD telemonitoring and the usability of the TECCU App. My name is … (Name of the facilitator) and I count on the assistance of … (Name of the assistant). We come from… (give a brief explanation of the participants´ affiliation).

Overview of the topic: A group of medical doctors from La Fe Hospital are developing a new web platform to manage remotely patients with IBD. They asked us to help them know if you agree with distance monitoring and what are your preferences about it. Moreover, you had the opportunity to use the platform demo and they need to know what you liked, what you didn´t like, or what you would change.

There are no wrong or right answers but differing points of view, so all your comments, positive or negative, will be interesting for us.

Ground rules: We are recording the session to do not miss any of your comments and transcribe them later. We won´t use any names that could identify you in our reports. Your comments will be used to plan future modifications in the TECCU app.

Do you have any questions? Well, let´s begin.

Question 1: What would you need from telemonitoring to improve the follow-up of your disease?

Question 2: What did you think about the TECCU app when you used it?

Question 3: Considering the design of the app, what did you like about it? What would you modify?

Question 4: What difficulties did you identify in the usability of the platform?

Question 5: Which aspects of your disease are well/badly addressed by the app?

Question 6: Do you think that the app improves the communication with your doctors or nurses? In what way?

Question 7: What elements of the app allow you to self-control your disease? Would they interfere with your usual activities?

Question 8: Could you tell us your opinion about the use of algorithms and action plans in the app?

Question 9: What would you need to feel safer using the app?

Question 10: Which were the contents of the app that you liked the most? How frequently would you access the educational resources and the telemonitoring program?

Question 11: (After a brief oral summary), is this an adequate summary?

Question 12: Have we missed anything that you would like to note?
